# Personality and willingness towards performance enhancement and body modification: A cross-sectional survey of a nationally representative sample of Norwegians

**DOI:** 10.3389/fspor.2022.906634

**Published:** 2022-12-22

**Authors:** Gunnar Breivik, Dominic Sagoe, Sigmund Loland

**Affiliations:** ^1^ Department of Sport and Social Sciences, Norwegian School of Sport Sciences, Oslo, Norway; ^2^ Department of Psychosocial Science, University of Bergen, Bergen, Norway; ^3^ Human Enhancement and Body Image Lab (HEBI Lab), Addiction Research Group, University of Bergen, Bergen, Norway

**Keywords:** human enhancement, body modification, bodily appearance, personality, big five

## Abstract

We conducted an exploratory investigation of the relationship between personality and willingness towards performance enhancement and body modification in Norway. The study is based on Norwegian Monitor data from a cross-sectional survey of a nationally representative sample of 4,233 (females: 49.9%) persons aged 15 to 96 (45.92 ± 18.02) years. Data were collected using a questionnaire containing demographic questions and measures of physical appearance satisfaction, physical activity level, personality (five-factor model), and willingness towards performance enhancement (e.g., substances that improve creative thinking) and body modification (e.g., use of muscle-building substances). Data were analyzed using descriptive statistics and multiple regression analyses. We found that 62.2% and 50.1% of our sample were either willing to use or contemplating using substances that reduce memory failure and enhance physical fitness respectively. Our sample was most willing or contemplating tattooing (30.0%) and generally skeptical of the other body modification methods with willingness to use or contemplating using substances to enhance muscularity least accepted (3.9%). Higher fantasy/openness and lower agreeableness were associated with higher willingness towards both performance enhancement and body modification. Additionally, higher extraversion and lower control/conscientiousness predicted higher willingness towards body modification. Our findings corroborate previous indications that performance enhancement and body modification are now mainstream. They also underline the importance of personality traits in willingness towards these practices.

## Introduction

### Human enhancement and body modification

Human enhancement refers to the improvement of human capacity, disposition and well-being through genetic, biomedical or pharmaceutical means in the absence of pathology or beyond what is necessary for sustenance or restoration of good health (1–3). Body modification on the other hand has been defined as permanent or semipermanent voluntary alteration of the human body that is not medically sanctioned such as plastic surgery, diet and exercise regimen, and other permanent and temporary cosmetic procedures (4).

Unlike human enhancement, there is contention regarding the definition of body modification. A distinction is made between body modification and nonmainstream body modification where the latter is defined (4) “as any permanent or semipermanent, voluntary alteration of the human body that is not medically mandated and that transgresses and challenges common assumptions and expectations of bodily presentation and/or aesthetic, and therefore may be considered extreme and/or deviant by members of mainstream Western society” (*p*. 4). Thus, tattoos, piercings, and plastic surgery for example, although often voluntarily done and permanent or semipermanent and normative in certain non-Western societies (e.g., male circumcision and female genital mutilation in some African and Middle Eastern societies) are not considered nonmainstream body modification. On the other hand, eccentrically placed, egregious, or extreme tattoos/piercings such as full-body or genital tattoos/piercings are regarded nonmainstream body modification. In the present study, “body modification” is used as an umbrella term for both (mainstream) body modification and nonmainstream body modification (4).

There is also contention regarding the conceptualization of human enhancement and body modification. On the premises that human enhancement is a category of body modification, every modification is arguably an enhancement, and that “body modification” unlike “human enhancement” is a normative and less prejudicial term, it has been argued and recommended that “body modification” be used as an umbrella term for both human enhancement and body modification (5). However, we distinguish between “human enhancement” and “body modification” in the present study.

The last few decades have witnessed advances in human enhancement and body modification. The widespread use of anabolic-androgenic steroids - AAS (6, 7) and various other human enhancement drugs (1, 8) as well as the proliferation of various body modification methods (9–11) for aesthetic and ergogenic enhancement is illustrative. It is noteworthy that the explosion of social media appears to promote and reinforce this phenomenon (12–15).

### Personality

Personality is defined as an organized and relatively stable set of psychological traits and mechanisms within an individual that influence the individual's interactions and adaptations to the physical and psychosocial environment (16). Personality is typically assessed as traits (17) defined as relatively stable cognitive, emotional and behavioral factors that establish individual identity and distinguishes people from others (18). The five-factor model (FFM) is a reliable, well-validated (19) and the foremost (16, 17) personality taxonomy. It consists of five personality factors or traits (neuroticism, extraversion, openness, agreeableness, and conscientiousness) with each associated with certain tendencies.

Neuroticism is defined in terms of the incidence and intensity of negative emotions and affect with higher scorers having a higher tendency towards anger, anxiety, depression, impulsivity, and vulnerability. Extraversion is defined as the magnitude and intensity of energy directed into the social world. Higher scores signal higher assertiveness, friendliness, sociability, and a higher tendency to experience positive emotions. Openness (also termed creativity, culture, fantasy, intellect, and openness to experience) is defined as aesthetic, emotional, intellectual and practical aptitude with higher scores signaling a higher appreciation of and interest in art and beauty, adventure, unusual ideas and values, new and diverse experiences, and intellectual curiosity. Agreeableness is defined in terms of compassion, cooperation, modesty, sympathy and trust with higher scores indicating a higher tendency to be altruistic, compassionate, cooperative, modest and sympathetic. Conscientiousness refers to the degree of efficiency, personal organization, and dependability with higher scores indicating a higher tendency to be organized, punctual and dependable, show self-discipline, aim for achievement, and prefer planned rather than spontaneous activity. These factors or traits are expatiated in the personality psychology literature (16, 17, 19).

### The relationship between personality, human enhancement and body modification

Personality traits have been implicated in human enhancement and body modification. The stimulation (20) theory of tattooing (21), extended to body modification, posits that body modification is an expression of high extraversion. An Austrian online survey (22) examined the link between personality and attitudes towards four human enhancement methods: pharmacological enhancement (e.g., use of psychostimulants such as modafinil to increase cognitive performance), current-based enhancement (e.g., deep brain stimulation to enhance memory and intelligence), genetic enhancement (human enhancement through genetic modification), and mind uploading (digital replication of the brain's cellular structure and upload onto an external storage medium for emulation.

In terms of the FFM, it was found that higher openness is associated with less negative attitudes towards pharmacological enhancement, genetic enhancement, and mind uploading. Additionally, it was found that attitudes towards human enhancement and body modification seems to differ by type with more negative attitudes towards pharmacological and genetic enhancement than current-based enhancement and mind uploading (22). In a similar study (23), agreeableness and conscientiousness correlated negatively with acceptance of human enhancement and body modification. Agreeableness and conscientiousness in particular correlated negatively with acceptance of genetic enhancement with conscientiousness having an additional negative correlation with deep brain stimulation. Additionally, multiple regression analysis showed that higher conscientiousness-industriousness is associated with lower acceptance of enhancement.

### Prevalence and correlates of human enhancement and body modification in Norway

Previous studies have examined the prevalence, attitudes and correlates of human enhancement and body modification in Norway. Historically, data from representative samples of Norwegians aged 18 to 69 from 1968 to 1989 showed a negative attitude towards drug use, high support for prohibition of drug use, and preference for harsher punishment for drug users (24). In our 2006 survey (25) of elite athlete (*n* = 234, males: *n *= 151) and general population (*n* = 428, males: *n *= 275) samples aged 18 to 35 years, the athlete sample showed higher physical appearance satisfaction than the general population sample. We also found that males have higher willingness towards performance enhancement whereas females have higher willingness towards body modification. In addition, whereas vitamins, nutritional supplements and hypoxic rooms were accepted by more than 65% of both samples, there was an almost unanimous rejection of erythropoietin (EPO), anabolic steroid and amphetamine use. Moreover, the athlete sample showed more reluctance towards performance enhancement and body modification than the general population.

In another 2006 survey (26) on body modification based on a representative sample of 1,862 northern Norway women aged 18 to 35 years, liposuction was the most prevalent body modification technique (25%), followed by breast augmentation (15%), rhinoplasty (7%) and abdominoplasty (5.6%). Also, whereas most respondents with interest in abdominoplasty showed no interest in other body modification procedures, most of the women with interest in breast augmentation and rhinoplasty expressed interest in more than one procedure. In terms of demographics, younger women univariately showed higher odds of interest in breast augmentation whereas lack of exercise was univariately associated with higher odds of interest in breast augmentation, liposuction, and abdominoplasty.

In the same study (26), results of multiple logistic regression analyses showed that lower educational attainment, and physical appearance evaluation are associated with higher odds of interest in breast augmentation, rhinoplasty, liposuction, and abdominoplasty whereas lack of exercise was associated with higher odds of interest in rhinoplasty. However, higher physical appearance orientation was associated with higher odds of interest in breast augmentation, rhinoplasty, and liposuction. On personality (FFM), lower conscientiousness, emotional stability and openness were univariately associated with higher odds of interest in breast augmentation, rhinoplasty, and liposuction whereas lower extraversion and agreeableness were univariately associated with higher odds of interest in rhinoplasty, and liposuction.

Additionally, a meta-analysis (27) of 32 studies shows that in the Nordic countries, Norway has the second-highest prevalence of AAS use (2.4%) after Sweden (4.4%). There is also experimental evidence from Norway (28) showing that AAS and EPO targets/users receive lower ratings on emotional stability, openness to experience and agreeableness. Also, in an online survey (29) of 15,654 (6,151 females) persons aged 16 to 91 years in Norway, the estimated prevalence of tattooing was 20.8% (females: 23.8%, males: 17.9%), of which 13.3% had readily visible tattoos. Results of multivariate logistic regression analysis indicated that females, being older than 19 years, higher body mass index, lifetime AAS use, and higher scores on extraversion were associated with higher odds of having a tattoo.

Furthermore, in the development of the Bergen Tanning Addiction Scale (30), based on an online sample of 23,537 (15,301 females, age range: 16–88, years mean = 35.8) respondents, being female and lower educational level were associated with higher tanning addiction. In terms of personality, higher neuroticism and extraversion, and lower intellect/openness were associated with higher tanning addiction.

### The present study

The present study is an exploratory investigation of the relationship between personality (FFM) and willingness towards performance enhancement (a specific form of human enhancement) and body modification in a nationally representative sample of Norwegians. Performance enhancement is operationalized in terms of use of substances that improve creative thinking, memory, physical fitness, workload/stress tolerance, physical strength and endurance, sexual ability, and emotional intelligence. Body modification is operationalized in terms of use of muscle-building substances, undergoing liposuction, facial plastic surgery, surgical operation for weight control, silicone implantation, tattooing, and substance use for youthful appearance. Since relatively little previous research could guide our expectations, instead of formulating specific hypotheses, we used an exploratory approach and formulated the following overarching research questions: 1) What is the prevalence of willingness towards performance enhancement and body modification in the Norwegian general population? 2) What is the relationship between personality traits (FFM) and willingness towards performance enhancement and body modification in the Norwegian general population?.

## Methods

### Data

The study is based on the 2015 Norwegian Monitor data collected by Ipsos Social Research Institute. Data were collected using telephone interviews (introductory questions) and a cross-sectional survey design. A simple random sampling from telephone directories was employed. Ipsos Group S.A. which conducts the Norwegian Monitor is a multinational market research company with ISO9001 and ISO202252 certification, and complies with the ESOMAR International Code on Market and Social Research. The Norwegian Monitor has approval from the Norwegian Data Protection Authority. Informed consent was obtained prior to data collection, and the dataset was anonymized before submission to the authors. The present study is based on data from a nationally representative sample of 4,233 (females: 49.9%) persons in Norway aged 15 to 96 (*M* = 45.92, *SD* = 18.02) years. The sample is weighted according to sex, age, and geographical region. Participant characteristics are presented in Table 1.

**Table 1:** Sample characteristics.

Variable	Frequency (*n*)	Percentage (%)
Sex (*N* = 4233)		
Female	2112	49.9
Male	2121	50.1
Age (range: 15–96), years (*N* = 4233)		
15–24	671	15.8
25–39	1042	24.6
40–59	1399	33.1
60–96	1122	26.5
Geographical region (*N* = 4233)		
Oslo and Akershus	1016	24.0
Other Eastern	1213	28.7
Western	1024	24.2
Central	582	13.8
Northern	399	9.4
Highest education (*N* = 4233)		
< university/college	1828	43.2
University/college	2401	56.8
Father's highest education (*N* = 3896)		
< university/college	2346	60.2
University/college	1550	39.8
Mother's highest education (*N* = 3944)		
< university/college	2576	65.3
University/college	1369	34.7
Income (*N* = 4027)		
< NOK 500,000 ≈ € 52,000	2602	64.6
≥ NOK 500,000	1426	35.4
Household income (*N* = 4044)		
< NOK 800,000 ≈ € 83,000	2227	55.1
≥ NOK 800,000	1817	44.9
Physical appearance satisfaction (*N* = 4159)		
Do not know	10	0.2
Very dissatisfied	108	2.6
Slightly dissatisfied	575	13.8
Neither satisfied nor dissatisfied	1181	28.4
Fairly satisfied	1982	47.7
Very satisfied	302	7.1
Physical activity level (*N* = 4233)		
Never	371	8.8
Less than once every 14 days	368	8.7
Once every 14 days	232	5.5
Once a week	662	15.6
Twice a week	905	21.4
3–4 times a week	1095	25.9
5–6 times a week	382	9.0
1 or more times per day	220	5.2

### Measures

Data were collected using a self-completion questionnaire.

#### Demographics

The questionnaire included questions assessing various demographic variables such as participants’ sex, age, highest education, father's highest education, mother's highest education, income, household income, and geographical region.

#### Physical appearance satisfaction

Physical appearance satisfaction was assessed using the question: “How satisfied or dissatisfied are you with your physical appearance?” Response options were: do not know (0), very dissatisfied (1), slightly dissatisfied (2), neither satisfied nor dissatisfied (3), fairly satisfied (4), and very satisfied (5). We therefore used an index score (range: 0–5) with higher scores denoting higher physical appearance satisfaction.

#### Physical activity level

Level of physical activity was assessed using the question: “How often would you say that you engage in physical activity in the form of training or exercise?” Response options were never (0), less than once every 14 days (1), once every 14 days (2), once a week (3), twice a week (4), 3–4 times a week (5), 5–6 times a week (6), and 1 or more times per day (7). We calculated an index score (range: 0–7) with higher scores denoting higher physical activity level.

#### Personality (FFM)

Personality was assessed using a 20-item (BFI-20) version (31) of the Big Five Inventory - BFI-44 (32). “I see myself as someone who: is depressed, blue; is outgoing, sociable; is original, comes up with new ideas; is helpful and unselfish with others; and does a thorough job” are example items assessing emotional stability, extraversion, fantasy/openness, agreeableness, and control/conscientiousness respectively. Items are answered on a five-point scale ranging from disagree strongly (1) to strongly agree (5). An index score was calculated for each factor/trait by summing participants’ responses on the corresponding items. In the present study, Cronbach's alphas were.74 for emotional stability,.80 for extraversion,.65 for fantasy/openness,.55 for agreeableness, and.53 for control/conscientiousness.

#### Willingness towards performance enhancement

Seven questions were used in assessing willingness to use various kinds of performance-enhancing substances: use of substances that improve creative thinking, memory, physical fitness, workload/stress tolerance, physical strength and endurance, sexual ability, and emotional intelligence. An example item is: “In the future, legal substances may be produced that may increase performance in various areas of life. How willing would you be to take the following substances if you at the same time ran a possible risk of a decrease in life expectancy?: substances that to a significant degree increase the ability to think creatively” (creative thinking). Items are answered on a four-point scale: cannot answer (0), not willing (1), may be willing (2), and willing (3). See Table 2. An index score was calculated by summing participants’ responses on the seven items. Total scores range between 0 and 21 with higher scores denoting more willingness to use of performance-enhancing substances. Cronbach's alpha across the seven questions was.86.

**Table 2:** Willingness towards performance enhancement (use of performance-enhancing substances).

Substances	Cannot answer	Not willing	May be willing	Willing
	*n* (%)	*n* (%)	*n* (%)	*n* (%)
Substances that to a significant degree increase the ability to think creatively (*N* = 4134)	255 (6.2)	3,014 (72.9)	703 (17.0)	162 (3.9)
Substances that reduce the failure of memory as a function of aging (*N* = 4141)	231 (5.6)	1,335 (32.2)	1,835 (44.3)	741 (17.9)
Substances that reduce the decrease in physical fitness as a function of aging (*N* = 4127)	250 (6.1)	1,809 (43.8)	1,622 (39.3)	447 (10.8)
Substances that increase the ability to tolerate workload/stress (*N* = 4125)	252 (6.1)	3,009 (72.9)	721 (17.5)	143 (3.5)
Substances that to a significant degree increase your physical strength and endurance (*N* = 4138)	250 (6.0)	3,030 (73.2)	707 (17.1)	151 (3.7)
Substances that to a significant degree increase your sexual ability (*N* = 4134)	297 (7.2)	2,727 (66.0)	938 (22.7)	172 (4.2)
Substances that give you better understanding of other people's emotions (*N* = 4128)	366 (8.9)	2,953 (71.5)	671 (16.3)	138 (3.3)

#### Willingness towards body modification

Willingness towards body modification was assessed using seven questions directly referring to different techniques and areas of body modification: use of muscle-building substances, undergoing liposuction, facial plastic surgery, surgical operation for weight control, silicone implantation, tattooing, and substance use for youthful appearance. An example item is: “In our society, it is possible to alter the appearance by different methods. How willing are you to use the following methods, even if they involve health risks?: take substances to get a muscular body” (muscle-building substances). Items are answered on a four-point scale: cannot answer (0), not willing (1), may be willing (2), and willing (3). See Table 3. An index score was calculated by summing participants’ responses on the seven items. Total scores range between 0 and 21 with higher scores denoting more willingness to resort to body modification techniques. Cronbach's alpha across the seven questions was.70.

**Table 3:** Willingness towards body modification.

Body modification	Cannot answer	Not willing	May be willing	Willing
	*n* (%)	*n* (%)	*n* (%)	*n* (%)
Take substances to have a muscular body (*N* = 4147)	39 (0.9)	3,948 (95.2)	128 (3.1)	32 (0.8)
Have liposuction to parts of your body you are dissatisfied with (*N* = 4145)	46 (1.1)	3,375 (81.4)	602 (14.5)	123 (3.0)
Have plastic surgery to alter your facial features (*N* = 4134)	45 (1.1)	3,648 (88.2)	361 (8.7)	80 (1.9)
Have a surgical operation to be able to eat what you want without adding weight (*N* = 4149)	59 (1.4)	3,673 (88.5)	314 (7.6)	103 (2.5)
Put silicone implants in your breasts or other places on your body to get a more attractive body (*N* = 4146)	125 (3.0)	3,676 (88.7)	236 (5.7)	109 (2.6)
Use tattooing on parts of your body (*N* = 4139)	50 (1.2)	2,848 (68.8)	686 (16.6)	555 (13.4)
Take substances to conserve a youthful appearance (*N* = 4142)	85 (2.0)	3,569 (86.2)	411 (9.9)	77 (1.9)

### Data analysis

We used descriptive statistics comprising frequencies and proportions as well as means and standard deviations to determine characteristics of the sample, and sample proportions in terms of willingness to use of performance-enhancing substances and body modification. Finally, we conducted multiple regression analyses to identify correlates of willingness to use performance-enhancing substances and body modification. Data analysis was conducted using SPSS version 28 (IBM Corp.).

## Results

### Prevalence of willingness towards performance enhancement

Sample proportions on willingness towards use of performance-enhancing substances are presented in Table 2. It is evident that 62.2% and 50.1% of our sample was either willing to use or contemplating using substances that reduce memory failure and enhance physical fitness respectively. Also, 26.9% of our sample was either willing to use or contemplating using substances that enhance sexual ability. Furthermore, 20.9% of our sample was either willing to use or contemplating using substances that enhance creative thinking, workload/stress tolerance, physical strength endurance, and emotional intelligence.

### Prevalence of willingness towards body modification

Table 3 presents sample proportions on willingness towards body modification. As shown in Table 3, our sample was most (30.0%) willing or contemplating tattooing. In addition, our sample was generally skeptical of the other body modification methods with willingness to use or contemplating using substances to enhance muscularity least accepted (3.9%). Except for willingness towards or contemplating liposuction (17.5%), the other methods (facial plastic surgery, surgical operation for weight control, silicone implantation, and substance use for youthful appearance) show about 10% percent willingness or contemplation.

### Correlates of both willingness towards performance enhancement and body modification

The models accounted for 8.1% and 18.8% of the variances in willingness towards performance enhancement and body modification respectively. See Table 4.

**Table 4:** Multiple regression analysis of predictors of willingness towards performance enhancement and body modification.

Variable	Performance enhancement				Body modification			
	*B*	*SE*	β	*t*	*B*	*SE*	β	*t*
Sex (female = 2, male = 1)	−0.81	0.12	−0.12	−6.72***	0.51	0.07	0.13	7.61***
Age (years)	−0.04	0.00	−0.19	−10.89***	−0.03	0.00	−0.30	−18.41***
Geographical region								
Other Eastern (Ref)								
Oslo and Akershus	0.05	0.15	0.01	0.32	0.06	0.08	0.01	0.70
Western	−0.09	0.15	−0.01	−0.60	0.06	0.08	0.01	0.73
Central	−0.06	0.18	−0.01	−0.32	0.17	0.10	0.03	1.68
Northern	−0.34	0.20	−0.03	−1.65	0.17	0.11	0.03	1.49
Highest education (university/college = 2, < university/college = 1)	0.18	0.12	0.03	1.46	−0.22	0.07	−0.05	−3.23**
Income (≥ NOK 500,000 = 2, < NOK 500,000) ≈ = 1 ≈ € 52,000	0.22	0.13	0.03	1.72	0.00	0.07	0.00	0.02
Physical appearance satisfaction	−0.25	0.06	−0.07	−4.06***	−0.39	0.03	−0.18	−11.18***
Physical activity level	0.03	0.03	0.02	0.98	−0.02	0.02	−0.02	−1.13
Personality								
Emotional stability	−0.02	0.01	−0.03	−1.53	−0.01	0.01	−0.02	−1.13
Extraversion	0.02	0.01	0.04	1.97	0.03	0.01	0.09	5.24***
Fantasy/openness	0.04	0.01	0.06	3.68***	0.02	0.01	0.05	3.17**
Agreeableness	−0.04	0.02	−0.04	−2.38*	−0.05	0.01	−0.09	−5.26**
Control/conscientiousness	−0.03	0.02	−0.03	−1.77	−0.02	0.01	−0.04	−2.08*

*R*^2^: performance enhancement = 8.0%, body modification = 18.9%.

* *p < *.05.

** *p < *.01.

*** *p < *.001.

#### Demographics and physical appearance

Males (*β* = −0.12, *p *< .001) had higher willingness towards performance enhancement whereas females (*β* = 0.13, *p *< .001) were associated with higher willingness towards body modification. Additionally, younger persons showed higher willingness towards performance enhancement (*β* = −0.19, *p *< .001) and body modification (*β* = −0.30, *p *< .001). Moreover, lower physical appearance satisfaction was related to higher willingness towards performance enhancement (*β* = −0.07, *p  *< .001) and body modification (*β* = −0.18, *p *< .001).

#### Personality

Higher fantasy/openness as well as lower agreeableness predicted higher willingness towards performance enhancement (fantasy/openness: *β* = 0.06, *p *< .001; agreeableness: *β* = −0.04, *p *< .05) and body modification (fantasy/openness: *β* = 0.05, *p *< .01; agreeableness: *β* = −0.09, *p *< .01).

### Correlates of only willingness towards body modification

#### Demographics

Persons with lower education (*β* = −0.05, *p *< .01) showed higher willingness towards body modification.

#### Personality

Higher extraversion (*β* = 0.09, *p *< .001) and lower control/conscientiousness (*β* = −0.04, *p *< .05) were associated with higher willingness towards body modification.

## Discussion

### Prevalences of willingness towards performance enhancement and body modification

The prevalences of willingness towards performance enhancement observed in the present study are similar to the observed prevalences from our 2006 Norwegian general population survey (25). Additionally, the prevalences of unwillingness towards body modification observed in the present study are similar to the prevalences from our 2006 Norwegian general population survey (25). In contrast, we noticed in juxtaposition, disparities between the two datasets with conspicuously lower estimates on muscularity, liposuction, tattooing and youthful appearance in the present study. Importantly, the present findings on the prevalence of willingness towards performance enhancement and body modification are elucidating and corroborate previous indications (1, 6, 8, 29, 33, 34) that these practices are now mainstream with a sizeable proportion of the population willing or contemplating engaging in these practices.

Given the skills and environmental constraints moderators of the link between intention and behaviour as delineated in the integrated behavioral model (35, 36), it is reasonable that the actual current prevalence (20.8%) of tattooing in Norway (29) is lower juxtaposed with the prevalence of willingness towards or contemplation of getting a tattoo (30.0%) observed in the present study. Indeed, we observed that our sample is generally more accepting of performance enhancement and relatively skeptical of the other body modification methods. A plausible explanation for this observation, based on the integrated behavioral model (35, 36) as posited above, is the relatively lower skills and environmental constraints such as self-administration moderating the relationship between intention and actual performance-enhancing substance use (e.g., pills for creative thinking and reduced memory failure) compared to the body modification techniques presented (e.g., liposuction and plastic surgery).

### Demographic and physical appearance correlates of willingness towards performance enhancement and body modification

Our finding associating males with higher willingness towards performance enhancement and females with higher willingness towards body modification is consistent with our previous finding (25). It is also in line with the preponderance of literature on gender/sex differences in performance enhancement (6, 7) and body modification (29, 30). Additionally, our finding that younger persons show higher willingness towards performance enhancement and body modification corroborates previous findings (26, 37) and the perspective that human enhancement is an element or fountain of contemporary youth culture and identity (1, 10, 38). Moreover, our finding that lower physical appearance satisfaction predicts higher willingness towards performance enhancement and body modification is tenable from an enhancement perspective. Furthermore, our finding that persons with lower education have higher willingness towards body modification is consistent with previous findings (26, 30).

### Personality and willingness towards performance enhancement and body modification

Our finding that higher fantasy/openness predicts higher willingness towards performance enhancement and body modification is consistent with previous findings from a survey of Canadian undergraduate students (39) and evidence from a recent Austrian online survey (22). This finding is reasonable given the aforementioned nomenclature (e.g., creativity, culture, fantasy, intellect), facets (aesthetic, emotional, intellectual and practical aptitude) and definition of openness in terms of appreciation of and interest in art and beauty, unusual ideas and values, new and diverse experiences, and intellectual curiosity (16, 19). Additionally, our finding that lower agreeableness predicted higher willingness towards performance enhancement and body modification is consistent with recent evidence from an Austrian survey indicating that agreeableness correlates negatively with acceptance of human enhancement (23). It also mirrors findings from a comparison of body-modified (tattoos and piercings) and non-modified persons (34, 37) and body piercing contemplators (40).

In line with the stimulation (20) theory of tattooing (21), and evidence that higher scores on extraversion predict higher odds of having a tattoo (29) or contemplating getting a body piercing (40), we found that higher extraversion is associated with higher willingness towards body modification. An explanation for this finding is that higher scores on excitement/sensation seeking, a facet of extraversion (19), have been associated with a higher tendency towards body modification (11, 39). Moreover, we found that lower control/conscientiousness is associated with higher willingness towards body modification in line evidence from a survey of American college students (34). As noted previously, persons with higher scores on conscientiousness tend to exhibit high self-discipline and preference for planned instead of spontaneous behaviour. Indeed, the deliberation facet of conscientiousness denotes a tendency towards critical consideration prior to behaviour (19). In this regard, this finding is tenable due to the spontaneity associated with body modifications such as tattoos and body piercings (41, 42).

### Implications for practice and future research

Our findings have clinical, policymaking, and technological implications. Consideration of the demonstrated mainstream proliferation of these practices in policymaking may be beneficial in averting potential future complications and harms emanating from nonmedical and illicit performance enhancement and body modification in the general population. Additionally, the correlates identified in the present study need consideration in the design and deployment of targeted preventive, treatment, and harm reduction interventions. Relatedly, the present findings may be informative for medical practitioners, technology providers, and regulatory authorities in the performance enhancement and body modification industry.

Our findings also have implications for future research. Future cross-disciplinary research, including philosophical, socio-cultural and ethical perspectives on human enhancement and body modification, may elevate the field. Experimental, mixed-methods and qualitative designs may also provide deeper insight and perspectives on human enhancement and body modification. The use of longitudinal designs may also elucidate trends in human enhancement and body modification in the general population. Furthermore, the use of measures of personality traits with facets, such as the NEO-PI-R (19), may provide an opportunity for facet-level exploration of the personality correlates of human enhancement and body modification. Similarly, the low explained variance in the regression models implies that future studies examine other potential correlates or confounders of performance enhancement and body modification such as participants’ exposure to these practices, and religious, ethical and moral attitudes. We also encourage psychometric effort in the development of well-validated measures of performance enhancement and body modification.

### Strengths and limitations

The present study is one of the few explorations of the prevalence and correlates of willingness towards performance enhancement and body modification in Norway. It improves on our previous study (25) by examining correlates of performance enhancement and body modification in a large nationally representative sample. It is one of the few studies to examine the association of personality traits with performance enhancement and body modification. However, the Cronbach's alpha value for openness to experience (.63) is relatively low (43) but acceptable as it is higher than the .60 cut-off score recommended for scales with few items (44) such as our personality (BFI-20) measure (31). Relatedly, the low Cronbach's alpha values for agreeableness (.55) and control/conscientiousness (.53) should be noted in the interpretation of the results on this factor.

Additionally, the two models explained only 8.0% and 18.9% of the variances in performance enhancement and body modification respectively. This implies that other unexamined variables such as participants’ familiarity with these practices, and religious, ethical and moral beliefs influence performance enhancement and body modification, and require exploration in future studies. Also, we are unable to make causal inferences due to our use of a cross-sectional survey design. Moreover, although self-reports have the advantage of eliciting data from many individuals in an ethical and relatively inexpensive manner, there is concern regarding the validity of reporting on a self-administered survey, especially when self-reports are not validated against objective criteria or data from other sources. Our personality measure also did not permit facet-level exploration of the five-factor model.

## Conclusion

The present exploratory study provides insight into the prevalence and correlates of willingness towards performance enhancement and body modification in the Norwegian population. It particularly elucidates the link between personality and willingness towards performance enhancement and body modification in the Norwegian population. Our findings corroborate previous indications that performance enhancement and body modification are now mainstream. They also underline the importance of personality traits in willingness towards performance enhancement and body modification. Our findings may be useful in the design and deployment of targeted preventive, treatment, and harm reduction interventions. Future interdisciplinary and multi-design research is needed to further highlight the practice of performance enhancement and body modification.

## Data Availability

The datasets presented in this article are not readily available because; The dataset is collected by a commercial research and survey agency and their property. However, it can be accessed in case of the need for check or control of the analyses in the article. Requests to access the datasets should be directed to; gunnarb@nih.no.

## References

[1] Evans-Brown M McVeigh J Perkins C Bellis MA. *Human Enhancement Drugs: The Emerging Challenges to Public Health.* North West Public Health Observatory (2012).

[2] Giubilini A Sanyal S. Challenging human enhancement. In: Clarke SJ Savulescu CA Coady J Giubilini A Sanyal S, editors. The ethics of human enhancement. Understanding the debate. Oxford: Oxford University Press (2016). p. 1–26.

[3] Juengst E Moseley D. *Human Enhancement*. Stanford Encyclopedia of Philosophy (2015). Accessed 05.07.2022 at: https://plato.stanford.edu/archives/sum2015/entries/enhancement/

[4] Thomas ML. Sick/beautiful/freak: nonmainstream body modification and the social construction of deviance. SAGE Open. (2012) 2:2158244012467787. 10.1177/2158244012467.

[5] Rembold S. Human enhancement’? It’s All about ‘body modification’! why we should replace the term ‘human enhancement’ with ‘body modification. NanoEthics. (2014) 8:307–15. 10.1007/s11569-014-0205-y.

[6] Sagoe D Molde H Andreassen CS Torsheim T Pallesen S. The global epidemiology of anabolic-androgenic steroid use: a meta-analysis and meta-regression analysis. Ann Epidemiol. (2014) 24:383–98. 10.1016/j.annepidem.2014.01.009. 24582699

[7] Sagoe D Pallesen S. Androgen abuse epidemiology. Curr Opin Endocrinol Diabetes Obes. (2018) 25:185–94. 10.1097/MED.0000000000000403. 29369917

[8] van de Ven K Mulrooney KJD McVeigh J. Human enhancement drugs. London: Routledge (2019).

[9] Featherstone M. Body modification. London, Thousand Oaks, New Delhi: SAGE (2000).

[10] Miller L. Deracialisation or body fashion? Cosmetic surgery and body modification in Japan. Asian Stud Rev. (2021) 45:217–37. 10.1080/10357823.2020.1764491.

[11] Wohlrab S Stahl J Kappeler PM. Modifying the body: motivations for getting tattooed and pierced. Body Image. (2007) 4:87–95. 10.1016/j.bodyim.2006.12.001. 18089255

[12] Andrew R Tiggemann AR Clark ML. Predicting body appreciation in young women: an integrated model of positive body image. Body Image. (2016) 18:34–42. 10.1016/j.bodyim.2016.04.003. 27240100

[13] Girard M Rodgers RF Chabrol H. Prospective predictors of body dissatisfaction, drive for thinness, and muscularity concerns among young women in France: a sociocultural model. Body Image. (2018) 26:103–10. 10.1016/j.bodyim.2018.07.001. 30041070

[14] Hilkens L Cruyff M Woertman L Benjamins J Evers C. Social media, body image and resistance training: creating the perfect ‘me’ with dietary supplements, anabolic steroids and SARM’s. Sports Med - Open. (2021) 7:81. 10.1186/s40798-021-00371-1. 34757466 PMC8579410

[15] Kuttschreuter M Hilverda F. Risk and benefit perceptions of human enhancement technologies: the effects of Facebook comments on the acceptance of nanodesigned food. Hum Behav Emerg Technol. (2019) 1:341–60. 10.1002/hbe2.177.

[16] Larsen R Buss D Wismeijer A Song J van den Berg S. Personality psychology. Domains of knowledge about human nature (3^rd^ edition). London: McGraw-Hill (2021).

[17] Matthews G Deary IJ Whiteman MC. Personality traits (3^rd^ edition). Cambridge: Cambridge University Press (2009).

[18] Holt N Bremner A Sutherland E Vliek M Passer MW Smith R. Psychology: The science of mind and behaviour (4^th^ edition). London, UK: McGraw Hill (2019).

[19] Costa PT Jr McCrae RR. NEO PI-R professional manual: revised NEO personality inventory (NEO PI-R) and NEO five-factor inventory (NEO FFI). Odessa, FL: Psychological Assessment Resources (1992).

[20] Eysenck HJ Eysenck SB. On the unitary nature of extraversion. Acta Psychol. (1967) 26:383–90. 10.1016/0001-6918(67)90034-0. 6082555

[21] Copes JH Forsyth CJ. The tattoo: a social psychological explanation. Int Rev Mod Sociol. (1993) 23:83–9.

[22] Grinschgl S Tawakol Z Neubauer AC. Human enhancement and personality: a new approach towards investigating their relationship. Heliyon. (2022) 8:e09359. 10.1016/j.heliyon.2022.e09359. 35574200 PMC9092989

[23] Schönthaler E Hofer G Grinschgl S Neubauer AC. Super-men and wonder-women: the relationship between the acceptance of self-enhancement, personality, and values. J Cogn Enhanc. (2022) 6:358–72. 10.1007/s41465-022-00244-9.

[24] Skretting A. Attitude of the Norwegian population to drug policy and drug offences. Addiction. (1993) 88:125–31. 10.1111/j.1360-0443.1993.tb02770.x. 8448503

[25] Breivik G Hanstad DV Loland S. Attitudes towards use of performance-enhancing substances and body modification techniques: a comparison between elite athletes and the general population. Sport Soc. (2009) 12:737–54. 10.1080/17430430902944183.

[26] Javo IM Sørlie T. Psychosocial characteristics of young Norwegian women interested in liposuction, breast augmentation, rhinoplasty, and abdominoplasty: a population-based study. Plast Reconstr Surg. (2010) 125:1536–43. 10.1097/PRS.0b013e3181d5135a. 20440172

[27] Sagoe D Torsheim T Molde H Andreassen CS Pallesen S. Anabolic-androgenic steroid use in the nordic countries: a meta-analysis and meta-regression analysis. Nord Stud Alcohol Drugs. (2015) 31:7–20. 10.1515/nsad-2015-0002.

[28] Sagoe D Huang K Molde H Andreassen CS Pallesen S. Perceived anabolic-androgenic steroid use is associated with perceived neuroticism. J Subst Use. (2016) 21:263–7.

[29] Sagoe D Pallesen S Andreassen CS. Prevalence and correlates of tattooing in Norway: a large-scale cross-sectional study. Scand J Psychol. (2017) 58:562–70. 10.1111/sjop.12399. 29105125

[30] Andreassen CS Pallesen S Torsheim T Demetrovics Z Griffiths MD. Tanning addiction: conceptualization, assessment and correlates. Br J Dermatol. (2018) 179:345–52. 29478244 10.1111/bjd.16480

[31] Engvik H Clausen S-E. Norsk kortversjon av big five inventory (BFI-20) [Norwegian brief version of big five inventory (BFI-20)]. Tidsskr Nor Psykol. (2011) 48:869–72.

[32] John OP Srivastava S. The big-five trait taxonomy: history, measurement, and theoretical perspectives. In: Pervin LA John OP, editors. Handbook of personality: theory and research (vol. 2). New York: Guilford Press (1999). p. 102–38.

[33] Sagoe D Pallesen S Dlova NC Lartey M Ezzedine K Dadzie O. The global prevalence and correlates of skin bleaching: a meta-analysis and meta-regression analysis. Int J Dermatol. (2019) 58:24–44. 10.1111/ijd.14052. 29888464

[34] Tate JC Shelton BL. Personality correlates of tattooing and body piercing in a college sample: the kids are alright. Pers Individ Differ. (2008) 45:281–5. 10.1016/j.paid.2008.04.011.

[35] Fishbein M. The role of theory in HIV prevention. AIDS Care. (2000) 12:273–8. 10.1080/09540120050042918. 10928203

[36] Fishbein M. A reasoned action approach to health promotion. Med Decis Making. (2008) 28:834–44. 10.1177/0272989X08326092. 19015289 PMC2603050

[37] Wohlrab S Stahl J Rammsayer T Kappeler PM. Differences in personality characteristics between body-modified and non-modified individuals: associations with individual personality traits and their possible evolutionary implications. Eur J Pers. (2007) 21:931–51. 10.1002/per.642.

[38] Bennett A Kahn-Harris K. After subculture: critical studies in contemporary youth culture. London: Palgrave (2004).

[39] Nathanson C Paulhus DL Williams KM. Personality and misconduct correlates of body modification and other cultural deviance markers. J Res Pers. (2006) 40:779–802. 10.1016/j.jrp.2005.09.002.

[40] Delazar ME. *The Relationship between Self-Esteem, Objectified Body Consciousness, Personality Traits and Body Modification: An Exploratory Study* (PhD thesis). Indiana, Pennsylvania: Indiana University of Pennsylvania (2004).

[41] Chen PJ. ‘It's the outline of a pig and then it has the words underneath “vegan for life”: vegans and their tattoos. Animal Studies Journal. (2020) 9:260–83. 10.14453/asj/v9.i2.11.

[42] Mensah E Inyabri I Mensah E. The discourse of tattoo consumption among female youth in Nigeria. Communicatio. (2018) 44:56–73. 10.1080/02500167.2018.1556222.

[43] Nunnally J. Psychometric methods (2nd ed). New York: McGraw-Hill (1978).

[44] Loewenthal KM. An Introduction to psychological tests and scales. Philadelphia, PA: Psychology Press (2001).

